# Chromosome identification and its application in Chinese hamster ovary cells

**DOI:** 10.1186/1753-6561-5-S8-O8

**Published:** 2011-11-22

**Authors:** Yihua Cao, Shuichi Kimura, Joon-young Park, Miyuki Yamatani, Kohsuke Honda, Hisao Ohtake, Takeshi Omasa

**Affiliations:** 1 Department of Biotechnology, Graduate School of Engineering, Osaka University, 2-1 Yamadaoka, Suita, Osaka 565-0871, Japan; 2 Institute of Technology and Science, The University of Tokushima, 2-1 Minamijosanjima-cho, Tokushima 770-8506, Japan

## Introduction

Chinese hamster ovary (CHO) cells [1] are today a very important host for the commercial-scale production of protein pharmaceuticals. Two sub clones of CHO cells, proline-requiring CHO K1 [2] and the dihydrofolate reductase (DHFR) gene-deficient CHO DG44 [3], are the most widely used for both scientific research and industrial applications [4][5,6]. Previously, we constructed a genomic bacterial artificial chromosome (BAC) library from mouse *Dhfr*-amplified CHO DR1000L-4N cell genome, which was provided 5-fold coverage of the CHO cell genome and analyzed the structure of amplicons of exogenous *Dhfr* amplification [7]. The BAC clones of this library could be landmarks for a physical map for CHO cell genome that are essential to the basic research and industrial application of CHO cell genome. In this study, we constructed the detail chromosomal physical map of CHO DG44 cell and investigated the chromosome rearrangements among CHO K1, DG44, and primary Chinese hamster cells. Moreover, to determine the effect of the palindrome structure on *Dhfr* amplification in CHO cells, we constructed three types of expression vectors with or without the junction region of the proposed amplicon and investigated the gene amplification and expression levels in transfected CHO DG44 cells.

## Materials and methods

### Cell lines, culture conditions, construction of vectors and transfection

CHO DG44, CHO K1 and primary Chinese hamster cells were used in this study. The primary Chinese hamster cells were isolated from lung tissue of 4 weeks old female Chinese hamster [7,8]. The structure of the Dhfr amplicon derived from CHO DR1000L-4N cells constructed from CHO DG44 cells was determined previously [7]. The structure has a large palindrome structure containing a small inverted repeat in the junction region. This small inverted repeat originates from the integrated vector. On the basis of this junction region, three expression vectors were constructed [8]. The pSV2-dhfr/GFP vector (vector A) was constructed from original vector [9] and GFP. The pcD-core region (vector B) was constructed from the core region (junction region containing two *Dhfr* copies and one *GFP*). The pcD-repeat free core region vector (vector C) was constructed from the repeat free core region (part of the junction region containing one *Dhfr* and one *GFP*). Three constructed plasmids were transfected into the CHO DG44 cells. In the *Dhfr*-amplification step, the transfected cells were cultivated with MTX at various concentrations of 50, 100, 250 and 500 nM.

### Fluorescence in situ hybridization using BAC clones as hybridization probes (BAC-FISH) and construction of CHO physical map

Chromosome spreads were prepared from exponential-phase cultures and BAC-FISH to chromosome spreads was carried out as described previously [7]. In brief, the BAC probes were detected using fluorescein isothiocyanate (FITC)-labeled streptavidin or an anti-DIG-rhodamine antibody. Chromosomes were counterstained with 4,6,-diamidino-2-phenylindole (DAPI) and observed under an Axioskop 2 fluorescence microscope. Photographs were taken with a CCD camera. After image processing was performed, the ImageJ software (http://rsbweb.nih.gov/ij/) was used to analyze the chromosomal loci of the BAC clone probes and the positions of the centromere on the chromosomes, and expressed as FLpter values [10].

## Results and discussion

### Construction of BAC-based physical map for Chinese hamster ovary cells

**Figure 1:**
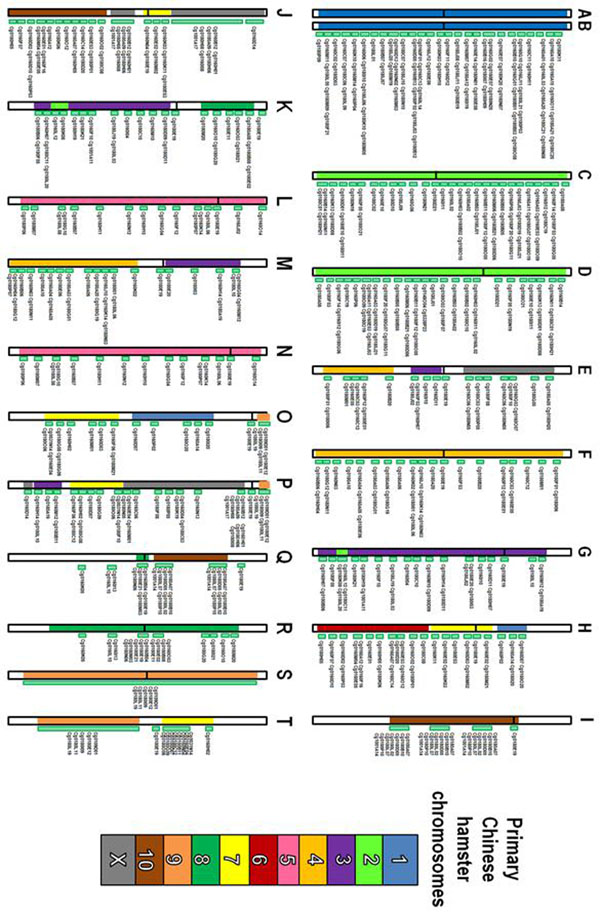
The physical map of CHO DG44 cells and karyotypic comparison to primary Chinese hamster cells on the basis of BAC-FISH results. Homologous region of CHO DG44 to Chinese hamster were colored according to the estimated Chinese hamster chromosomes.

A CHO genomic BAC library consisted of 122,281 clones was constructed in a previous study [7]. Three hundred BAC clones were randomly selected from this library and mapped onto each chromosome of CHO DG44 cells by BAC-FISH. The FISH signal location of BAC clone probes on each chromosome were determined by digital image analysis and expressed as FLpter values. The 185 BAC clones were also mapped on the chromosomes of CHO K1 cell line and 94 clones on primary Chinese hamster chromosomes to investigate the chromosome rearrangements. The karyotypic comparison between CHO DG44 and primary Chinese hamster cells was diagrammatically summarized in Fig. 1. The 20 chromosomes in CHO DG44 cell line were aligned in order of decreasing size and assigned letters from A to T. The normal Chinese hamster chromosome number were estimated using BAC-FISH, end-sequencing and previous comparative study between mouse and Chinese hamster [11]. The chromosomes A, B, C, F, L, N, R and S were derived from normal Chinese hamster chromosomes without large rearrangements, and then these chromosomes were conserved between CHO DG44 and K1 cells (data not shown). This result suggested that these chromosomes were very stable and essential in CHO cells and supposedly conserved in other CHO cell lines.

### FISH analysis of gene-amplified chromosomal region of transfected cells [8]

The chromosomal site of integration of a transgene affects its transcription rate. This phenomenon is called a positive effect and is often observed in transgenic organisms in which the transcription of an inserted transgene is affected by the proximity of the transgene to heterochromatin [12]. To understand the effect of structure of *Dhfr* amplicon on the chromosomal site of integration, we performed two-color BAC-FISH analysis for transgene. In our previous study, we determined that the amplified gene is integrated in one specific chromosome (chromosome O). The constructed vectors B and C contain the palindrome structure obtained from the BAC clone Cg0031N14 located on chromosome O. The summarized results of integrated chromosomal sites under various MTX concentrations are shown in Table 1. Interestingly, the transfected cells whose transgene is located at the same chromosomal position of the BAC clone Cg0160E04 were abundantly observed in the vector B and C integrations. It is likely that the chromosomal position of the BAC clone Cg0160E04 on chromosome O is a hot spot for *Dhfr* amplification in CHO cells.

**Table 1:** Ratios of amplified genes located at same position of BAC clone Cg0160E04 on chromosome O.

MTX concentration (nM)	Vector A (%)	Vector B (%)	Vector C (%)
50	0 (0/14) ^a^	0 (0/11) ^a^	0 (0/15) ^a^
100	0 (0/10) ^a^	33.3 (4/12) ^a^	18.2 (2/11) ^a^
250	0 (0/10) ^a^	61.5 (8/13) ^a^	57.1 (8/14) ^a^
500	0 (0/10) ^a^	70.8 (17/24) ^a, b^	66.7 (6/9) ^a^

^a^ (Number of chromosomes detected at same position of BAC clone Cg0160E04 on chromosome O/total number of analyzed chromosomes). ^b^ Further results were obtained from the previous study [8].

In summary, we constructed a BAC-based physical map for CHO DG44 cells and analyzed genome-wide rearrangements of chromosome among CHO cells. This BAC-based physical map will greatly facilitate the studies of CHO cell genome. The BAC clones comprising this physical map could also provide a genome-wide resource for analysis of chromosome rearrangements, chromosome structure, comparative genome hybridization, gene targeting, and functional genomics.
